# Thermal heterogeneity is an important factor for maintaining the genetic differentiation pattern of the pelagic barnacle *Lepas anatifera* in the northwest Pacific

**DOI:** 10.1002/ece3.9843

**Published:** 2023-02-22

**Authors:** Xiao‐Nie Lin, Li‐Sha Hu, Zhao‐Hui Chen, Yun‐Wei Dong

**Affiliations:** ^1^ The Key Laboratory of Mariculture, Ministry of Education, Fisheries College Ocean University of China Qingdao China; ^2^ Function Laboratory for Marine Fisheries Science and Food Production Processes Pilot National Laboratory for Marine Science and Technology Qingdao China; ^3^ Frontier Science Center for Deep Ocean Multispheres and Earth System (FDOMES) and Physical Oceanography Laboratory Ocean University of China Qingdao China; ^4^ Qingdao National Laboratory for Marine Science and Technology Qingdao China

**Keywords:** distribution pattern, *Lepas*, Northwest Pacific, open ocean, pelagic barnacle, population differentiation

## Abstract

Macrobenthic invertebrates are ubiquitously distributed in the epipelagic zone of the open ocean. Yet, our understanding of their genetic structure patterns remains poorly understood. Investigating the genetic differentiation patterns of pelagic *Lepas anatifera* and clarifying the potential roles of temperature maintaining this pattern are crucial for our understanding of the distribution and biodiversity of pelagic macrobenthos. In the present study, mitochondrial cytochrome oxidase subunit I (mtDNA COI) from three South China Sea (SCS) populations and six Kuroshio Extension (KE) region populations of *L. anatifera* sampled from fixed buoys and genome‐wide SNPs from a subset of populations (two SCS populations and four KE region populations) were sequenced and analyzed for investigating the genetic pattern of the pelagic barnacle. Water temperature was different among sampling sites; in other words, the water temperature decreased with latitude increases, and the water temperature on the surface was higher than in the subsurface. Our result showed that three lineages with clear genetic differentiation were found in different geographical locations and depths based on mtDNA COI, all SNPs, neutral SNPs, and outlier SNPs. Lineage 1 and lineage 2 were dominant in the subsurface populations and surface populations from the KE region, respectively. Lineage 3 was dominant in the SCS populations. Historical events during the Pliocene epoch shaped the differentiation of the three lineages, while, nowadays, temperature heterogeneity maintains the current genetic pattern of *L. anatifera* in the northwest Pacific. The subsurface populations were genetically isolated from the surface populations in the Kuroshio Extension (KE) region, implying small‐scale vertical thermal heterogeneity was also an important factor maintaining the genetic differentiation pattern of the pelagic species.

## Graphical abstract

Macrobenthic invertebrates are ubiquitously distributed in the open oceans; however, we are rarely understanding their phylogeographic patterns in the open ocean. In the present study, we investigated the phylogeographic pattern of *Lepas anatifera* attached to fixed buoys and moorings set in the Northwest Pacific. Our results showed that there were three genetic‐differentiated lineages in different geographical locations and depths and confirmed that both horizontal and vertical thermal heterogeneity was important for maintaining the genetic differentiation of *L. anatifera* populations in the open oceans.

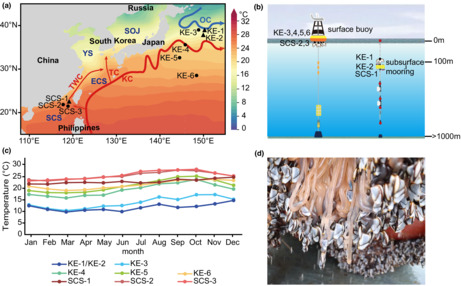

## 1 INTRODUCTION

Macrobenthic invertebrates are ubiquitously distributed in the epipelagic zone of the open ocean (Carlton et al., 2017), while we are rarely understanding their phylogeographic patterns there. Phylogeographic studies provide critical insights into the history of population divergence (Avise, 2000) and further improve our understanding of how environmental change impacted the distribution and diversity of the species (Zhang et al., 2019). Especially in the open ocean, where numerous species are highly sensitive to global warming, exploring the role of temperature shaping the phylogeographic patterns can enhance our understanding of evolutionary processes and our ability to predict species' responses to environmental change (Pinsky et al., 2019). Macrobenthic invertebrates, due to their wide distribution, long residency in specific habitats, and high sensitivity to changes in various environmental conditions (Mohan et al., 2013), are ideal species to investigate their phylogeographic patterns and clarify the potential drivers.

Geographical differentiation is common for marine species due to historical events (Palumbi, 1994; Wang et al., 2015), oceanographic forces (Chan et al., 2022; Dong et al., 2012; Li, Dai, et al., 2021), habitat discontinuity (Wort et al., 2019), dispersal ability (Cowen & Sponaugle, 2009), and water temperature (Cheng & Sha, 2017). In the open sea, environmental gradients play an important role in genetic divergence (Hudson et al., 2017); for example, large‐scale temperature heterogeneity can lead to local adaptation and genetic differences (Saeedi et al., 2019; Sasaki & Dam, 2019), implying that genetic divergence at depth gradients contains important information for the understanding of phylogenetic relationships and evolutionary history (Behrens et al., 2021; Stefánsson et al., 2009). Therefore, phylogeographic studies conducted in both vertical and horizontal directions might deepen our understanding of the genetic divergence or species evolutionary history of marine species.

The goose barnacles of the genus *Lepas* were considered ideal candidates to investigate population differentiation in the open ocean due to their cosmopolitan distribution (Schiffer & Herbig, 2016). *L. anatifera* is the goose barnacle with the highest abundance worldwide (Young, 1990). They have been reported from various floating objects including plastics (Aliani & Molcard, 2003), pumice stones (Coombs & Landis, 1966), woods (Nilsson‐Cantell, 1930), and drifting kelp rafts (Hobday, 2000). Their planktonic larval stage can take up to 2 months (Anderson, 1994; Darwin, 1851; Hinojosa et al., 2006), thus enabling long‐distance dispersal. A previous study of *L. anatifera* throughout its wide distribution range provided a global view of its genetic differentiation pattern, revealing three major phylogenetic groups (Schiffer & Herbig, 2016).

In the Northwest Pacific (NWP), there are complex oceanographic current systems, including the Kuroshio Current (KC), Kuroshio Extension Current (KEC), and Oyashio Current (OC). The northward Kuroshio transported a large amount of heat from low latitudes to middle and high latitudes in a very narrow area, and the southward hydrophile transported low temperature and low salt water from high latitudes to low latitudes, resulting in a significant ocean temperature gradient in the Kuroshio Extension region (Qiu, 2001). Under the context of global warming, the Kuroshio is being intensified (Chen et al., 2019), and clarifying the changes in biodiversity and distribution of marine species affected by the Kuroshio will provide us with a better understanding of the impact of climate change on marine species. To identify and quantify the dynamic and thermodynamic processes governing the variability and the interaction between the Kuroshio Extension and the recirculation gyre, the inverted echo sounders with bottom pressure gauges and current meters, subsurface moorings, surface buoys, and dozens of Argo profiling floats have been set in the Kuroshio Extension region (Zhu et al., 2021). *Lepas anatifera* has been ubiquitously found on the surfaces of these buoys and subsurface moorings.

The pelagic barnacles *L. anatifera* on these moorings and buoys are important for studying the genetic diversity and the factors maintaining the genetic differentiation pattern in the NWP. In the present study, we focus on the genetic differentiation patterns of *L. anatifera* sampled vertically and horizontally from these buoys and subsurface moorings in both the South China Sea (SCS) and KEC. We aimed to investigate the following questions: (1) whether there is genetic divergence existing among NWP populations for *L. anatifera*; (2) if there is, what is the role of temperature driving the formation and maintenance of the corresponding pattern? Our results provide new insights into the mechanisms of the formation and maintenance of species' genetic patterns in the NWP. Moreover, our research approach is of general relevance for future studies on phylogeographic patterns and genetic adaptation of species distributed in the epipelagic zone of the open oceans.

## 2 MATERIALS AND METHODS

### 2.1 Sampling and sequencing

**FIGURE 1:**
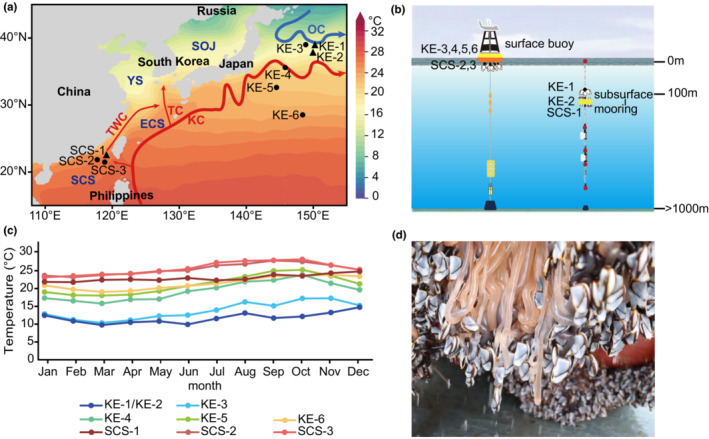
Sampling sites of *Lepas anatifera* populations in the Northwest Pacific and water temperatures of corresponding sites. (a) Sampling sites of *L. anatifera* populations. Circles represent the surface sampling sites, and squares represent the subsurface sampling sites. SCS, South China Sea; ECS, East China Sea; YS, Yellow Sea; SOJ, Sea of Japan; KC, Kuroshio current; TWC, Taiwan current; TC, Tsushima current; OC, Oyashio current; (b) Sketch map of surface buoys and subsurface moorings; (c) Average water temperature from 2005 to 2017 in the sampling sites. The temperature of KE‐1 and KE‐2 were almost the same; (d) In situ photo of *L*. *anatifera* attached to the buoys and moorings.

A total of 186 *L*. *anatifera* were collected from three sites in the South China Sea (SCS) and six sites in the Kuroshio Extension (KE) region (Figure 1a, Table 1). Among all these sites, samples in KE‐1, KE‐2, and SCS‐1 were sampled in the subsurface moorings (water depth > 100 m), and all the other sites were sampled in the fixed surface buoys (water depth < 50 m) (Figure 1b).

**TABLE 1:** Sampling sites and diversity indices for each sampling site of *Lepas anatifera*.

Location	Site	COI				GBS‐SNPs			
		Nc	*n*	*h* (mean ± SD)	*π*_c_ (mean ± SD)	Ns	*H*_e_	*H*_o_	*π*_G_
Kuroshio Extension (KE) region	KE‐1	21	17	0.967 ± 0.030	0.01496 ± 0.00131				
	KE‐2	21	20	0.995 ± 0.016	0.05241 ± 0.01530				
	KE‐3	15	15	1.000 ± 0.024	0.06554 ± 0.01800	17	0.05676	0.04786	0.05962
	KE‐4	22	21	0.996 ± 0.015	0.06315 ± 0.01470	20	0.06179	0.05318	0.06448
	KE‐5	20	20	1.000 ± 0.016	0.06984 ± 0.01464	19	0.06447	0.04630	0.06675
	KE‐6	24	22	0.993 ± 0.014	0.08929 ± 0.01074	20	0.06356	0.05506	0.07034
South China sea (SCS)	SCS‐1	23	22	0.996 ± 0.014	0.01127 ± 0.00101	20	0.06745	0.07909	0.07064
	SCS‐2	22	19	0.987 ± 0.018	0.00847 ± 0.00075				
	SCS‐3	22	21	0.996 ± 0.015	0.00886 ± 0.00062	20	0.06701	0.07798	0.00193
	Total	190	157	0.9970 ± 0.0011	0.09796 ± 0.00138	116			

*Note*: Nc, number of sequences; *n*, number of haplotypes; *h*, haplotype diversity; *π*_c_, nucleotide diversity of COI. Ns, number of individuals for GBS‐seq; *H*_e_, expected heterozygosity; *H*_o_, observed heterozygosity; *π*_G_, nucleotide diversity of GBS‐SNPs.

For sequencing mitochondrial cytochrome oxidase subunit 1 (COI), the total genomic DNA of the 186 *L*. *anatifera* samples was extracted from muscles either from the peduncle or the base of the cirri using a modified cetyltrimethylammonium bromide (CTAB) method (Ding et al., 2018). The universal primers LCO1490 and HCO219879 (Folmer et al., 1994) and the primers jgHCO2198 and jgLCO1490 (Geller et al., 2013) were used for polymerase chain reaction (PCR). The forward and reverse sequences were assembled using SeqMan application of DNAStar software (SeqMan NGen®. Version 17.2. DNASTAR.). Multiple sequence alignment was then conducted using the plug‐in Clustal W program in MEGA X (Kumar et al., 2018) with default parameter settings.

A total of 116 samples were selected for Genotyping‐by‐Sequencing (GBS). DNA was extracted with an SDS (sodium dodecyl sulfate) protocol, and DNA libraries were constructed using NEBNext DNA Library Prep Kits (New England Biolabs). Samples were processed and sequenced using Illumina HiSeq 2500 Rapid Run flow cell (150 base pairs paired‐end reads) at Novogene (Beijing, China). The quality of the sequence data was assessed with fastp software (Chen et al., 2018). The genome of *L. anserifera* (Cirripedia, Lepadidae) (Ip et al., 2021) was used as a reference genome and clean data were aligned to the genome with BWA (Li & Durbin, 2010). SNP calling was conducted using Samtools (Danecek et al., 2021) and the outputs were put into gstacks program (−‐rm‐unpaired‐reads) in Stacks to build loci and identify SNPs by incorporating paired‐end reads. The populations module (−*r* 0.6, −‐filter‐haplotype‐wise) was used to filter the data. We retained only those loci that were genotyped in at least 60% of samples in a population and unshared SNPs were pruned to reduce haplotype‐wise missing data. VCFtools (Danecek et al., 2011) was used to further filter the SNPs with the following settings: minimum minor allele frequency (−‐maf 0.01), the proportion of missing data (−‐max‐missing 0.2), mean depth values (−‐min‐meanDP 3), exclusion all genotypes with a quality below 20 (−‐minGQ 20) and inclusion of only bi‐allelic sites (−‐min‐alleles 2 ‐‐max‐alleles 2).

### 2.2 Genetic diversity and phylogenetic analyses

Molecular diversity for COI was estimated by the number of haplotypes (*n*), haplotype diversity (*h*), and nucleotide diversity (*π*_c_) for each population using DnaSP 6.0 (Rozas et al., 2017). For GBS‐seq, expected heterozygosity (*H*_e_), observed heterozygosity (*H*_o_), and nucleotide diversity (*π*_G_) were calculated based on filtered SNPs mentioned above using the populations program in Stacks v.2.5.1 (Rochette et al., 2019).

Neighbor‐Joining (NJ) tree was constructed in MEGA X using the COI sequences of 186 individuals with 1000 bootstrap replicates. To further verify the reliability of phylogenetic tree topology based on COI, SNPs filtered from the GBS (including the entire SNP dataset, the neutral dataset, and the outlier dataset, see below for the details of identifying the neutral and outlier loci) were used to perform phylogenetic analyses under the same settings described above. To better understand the relationships between the lineages in a global context, we also construct an NJ tree using COI sequences of *L. anatifera* from global samples (GenBank accessions: GU993588‐GU993597 for samples in Chile, GU993603‐GU993612 for samples in Spain, GU993619‐ GU993628 for samples in the Gulf of Mexico; Schiffer & Herbig, 2016) and the partial COI sequences obtained from the present study. The mean distances between lineages of different sites as indicated by the phylogenetic analyses were calculated in MEGA X (Kumar et al., 2018).

To get a clear genealogical relationship among haplotypes, a minimum‐spanning network (MSN) was constructed by PopART (Leigh et al., 2015) based on COI haplotypes.

### 2.3 Historical dynamics analysis

For COI, the neutrality test of Tajima's *D* (Tajima, 1989) and Fu's *F*_S_ (Fu & Li, 1993) and mismatch distribution were calculated using Arlequin 3.1 (Excoffier et al., 2005). Significant negative neutrality test values suggest that these populations may have experienced expansion. We tested the goodness of fit of the actual distributions with the expected distributions under a sudden expansion model by calculating the sum of squared deviations (SSD) and Harpending's raggedness index (HRI) following 1000 coalescent simulations. Nucleotide mismatch distribution plots can also indicate the historical demography of populations: a single peak indicates that the population has experienced an expansion in size or distribution, and multiple peaks indicate that the populations are in equilibrium. In addition, Bayesian skyline plots (BSPs) were conducted in BEAST v2.6.6 (Bouckaert et al., 2014) to detect past demographic changes in effective population size for each lineage present in the phylogenetic tree; 100 million MCMC steps sampled every 1000 generations were performed under the assumption of a Relaxed Clock Log‐Normal and the nucleotide substitution model inferred with MEGA X. The first 10% of the trees were discarded as the burn‐in. Tracer v1.7.2 (Rambaut et al., 2018) was used to check the convergence to the stationary distribution and sufficient effective sampling sizes for each estimated parameter. The results of the three repetitive operations were put into LogCombiner 2.6.6 (Bouckaert et al., 2014) to construct the final BSP for each lineage.

The mutation rate of COI estimated for crustacean species ranges from 1.4% to 2.33% per million years (Knowlton & Weigt, 1998; Schubart et al., 1998) and was used to estimate divergence time between lineages according to the K2P genetic distance calculated in MEGA X (Kumar et al., 2018). Besides, we used 1.865% as the average substitution rate to estimate the expansion time of each lineage based on the formula *τ* = 2*μ*kt (Slatkin & Hudson, 1991), where *τ* is a population expansion parameter in mismatch distribution, *μ* is the average nucleotide substitution rate, and k is the numbers of nucleotides.

### 2.4 Detection of genetic differentiation associated with temperature

To detect loci underlying local adaptation, a Bayesian approach as implemented in BAYENV2 (Coop et al., 2010; Günther & Coop, 2013) was applied to the entire SNPs dataset. The Bayesian approach considers the effect of population structure, using a covariance matrix based on neutral SNPs to control for demographic effects when testing for correlations between environmental factors and genetic differentiation (Coop et al., 2010). To identify the neutral loci, we first excluded the candidate loci under natural selection detected in BayeScan 2.1 (Foll & Gaggiotti, 2008).

BayeScan decomposes locus‐population *F*_ST_ coefficients into a population‐specific component (beta), shared by all loci and a locus‐specific component (alpha) shared by all the populations using a logistic regression (Foll & Gaggiotti, 2008). Loci with a positive value of alpha were considered to be under divergent selection. Prior odds of 100 were used for identifying the candidates of the selected loci, and then BayeScan was run with the settings of 100,000 iterations, a thinning interval of 10, 20 pilot runs of 5000 iterations each with a burn‐in length of 50,000. Loci with a false discovery rate (FDR) of 0.05 were considered under selection. Then, we retained the SNPs within Hardy–Weinberg equilibrium (HWE) at *p* < .005 and further pruned the SNPs for linkage disequilibrium (LD) using PLINK (Purcell et al., 2007) at a thread of 0.2. The resulting SNPs were determined as “neutral SNPs.” To ensure that the estimated matrix was convergent, four independent runs with different random seeds were run based on neutral loci. The average water temperatures in January (T1) and August (T8) were selected as the cold and warm months, respectively. As environmental factors that may constrain the survival and reproduction of *L. anatifera* (Patel, 1959), these temperature variables were tested for association with genetic variation. Each environmental parameter was standardized by subtracting the mean and dividing it by the standard deviation of the parameter across all sampling sites. To reduce stochastic errors, the detection of environmental correlation was also run three times independently with different random seeds. SNPs with log_10_ Bayes factor (BF) >1.5 (Jeffreys, 1961) in the results were identified as loci strongly associated with environmental variables. Candidate genes containing selected SNPs were functionally annotated with the ANNOVAR software (Wang et al., 2010).

To estimate the degree to which the genomic variations among different populations can be explained by temperature heterogeneity and to test the reliability of temperature‐related outliers detected using BAYENV2, redundancy analysis (RDA) was performed based on adaptive data sets associated with temperature using the RDA function from the *vegan* package (Oksanen et al., 2013). Analyses of variance (ANOVAs) were performed to check the RDA model for significance and marginal ANOVAs (999 permutations) were run to determine whether the two temperature variables were significantly correlated with genetic variations.

### 2.5 Population structure

Genetic differentiation between populations was represented by pairwise *F*_ST_ values (Weir & Cockerham, 1984), which were estimated based on COI data using Arlequin 3.1 (Excoffier et al., 2005) with 1000 permutations performed to test for significance.

An analysis of molecular variance (AMOVA) (Excoffier et al., 1992) was also conducted based on COI data to estimate population structure among putative groupings. All individuals were divided into three groups according to the sampling latitude and depth: Group1 (high‐latitude subsurface group: KE‐1 and KE‐2), Group 2 (high‐latitude surface group: KE‐3, KE‐4, KE‐5, and KE‐6), and Group 3 (low‐latitude group: SCS‐1, SCS‐2, and SCS‐3). Significance was tested with 1000 permutations.

Isolation by distance (IBD) model is often used to test whether the migration of populations conforms to the stepping‐stone model (Bohonak, 2002). If the population conforms to the IBD model, the genetic distance between populations [*F*_ST_/(1‐*F*_ST_)] is linearly correlated with geographic distance. The correlation between geographic distance and genetic distance was detected using the Mantel test in GenAlEx 6.5 (Peakall & Smouse, 2012) after the geographic distance (GGD) and genetic distance matrix (GD) of COI and neutral SNPs were generated.

Population structure and individual ancestry were predicted based on all SNPs, neutral SNPs, and temperature‐related outliers, respectively, using ADMIXTURE v.1.3.0 (Alexander et al., 2009), which implemented a maximum likelihood (ML) estimation method. The number of genetic groups represented by *K* values was predefined from one to nine. The optimal *K* values were assessed using the cross‐validation (CV) procedure implemented in ADMIXTURE with the ‐‐cv flag. The principal component analysis (PCA) using the R package tidyverse was further applied to investigate the genetic divergence among all individuals.

## 3 RESULTS

### 3.1 Genetic diversity and population structure revealed by the COI

With mtDNA COI as the genetic marker, 157 haplotypes were identified (GenBank Accession numbers: OP215356‐OP215545) from 186 individuals attached to subsurface moorings or surface buoys (Figure 1d). Duplicated COI haplotypes were found in four individuals. All populations of *L. anatifera* were identified with high haplotype diversity (0.997 ± 0.001) and nucleotide diversity (0.098 ± 0.001) with overall value. The nucleotide diversity values of populations in the KE region (COI: 0.015–0.089) were generally higher than that of the SCS populations (COI: 0.008–0.011) (Table 1).

**FIGURE 2:**
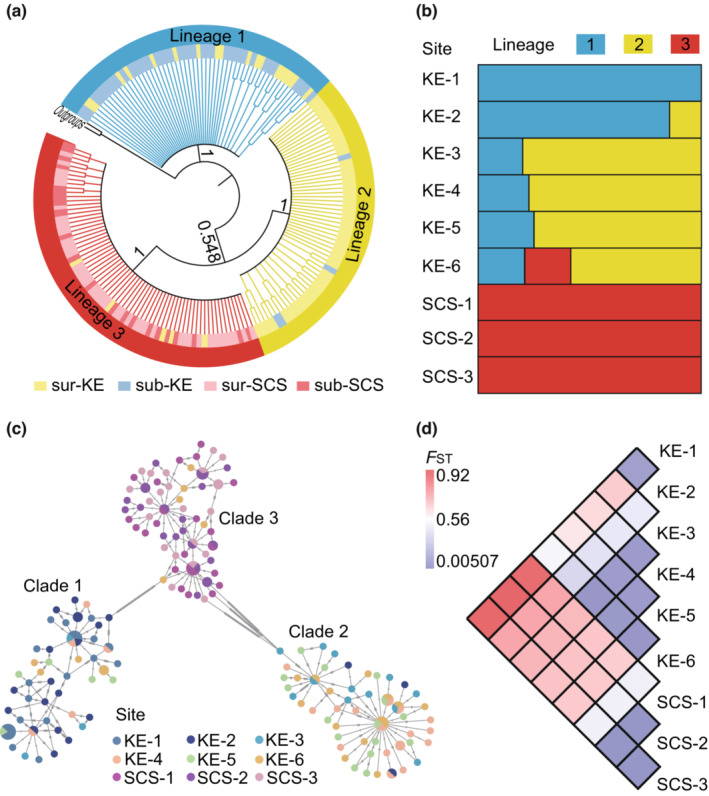
Phylogeographic structure of *Lepas anatifera* populations in Northwest Pacific based on mtDNA COI. (a) Neighbor‐Joining tree of *L. anatifera* genetic lineages based on COI; (b) The distribution patterns of each lineage in the sampling sites; (c) Minimum spanning network (MSN) constructed from haplotypes of COI sequences, each dot represents a haplotype; (d) *F*_ST_ values between each population pair. sur‐KE, surface sites in the Kuroshio Extension (KE) region; sub‐KE, subsurface sites in the Kuroshio Extension (KE) region; sur‐SCS, surface sites in the South China Sea (SCS); sub‐SCS, subsurface sites in the South China Sea (SCS).

**FIGURE 3:**
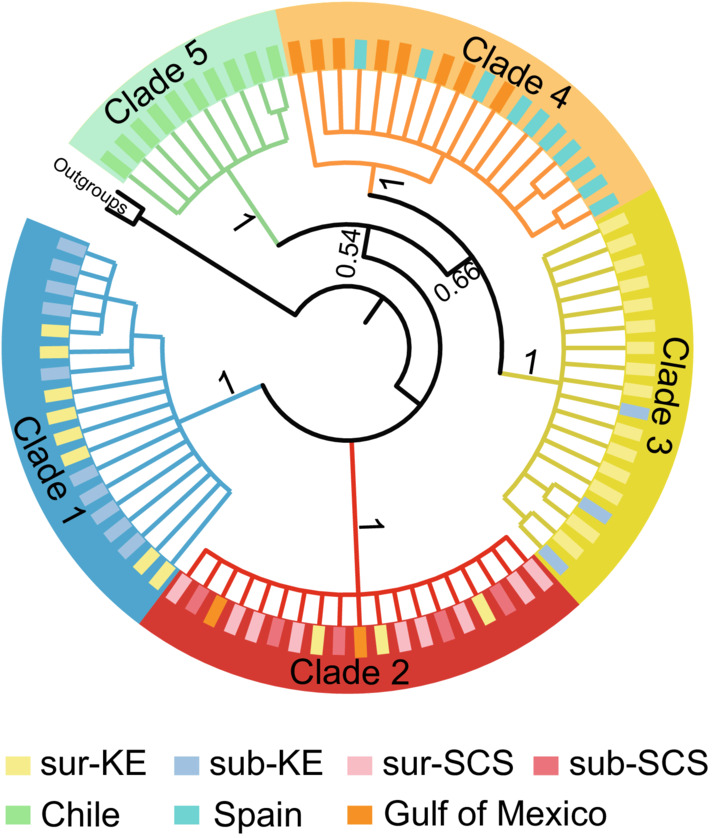
Neighbor‐Joining tree of *Lepas anatifera* based on global COI data. Clade 1 and Clade 3 (e.g., lineage 1 and lineage 2 obtained in the present study) were unique in Northwest Pacific, Clade 2 (e.g., lineage 3) included two sequences from Gulf of Mexico in addition to the South China Sea individuals. Clade 4 included sequences from Spain and the Gulf of Mexico, and Clade 5 included sequences from Chile.

NJ tree constructed based on COI revealed three diverged lineages in *L. anatifera* populations. Among them, lineage 2 and lineage 3 clustered together and then clustered with lineage 1 (Figure 2a). Individuals in each lineage presented a clear population‐specific pattern (Figure 2b). Lineage 1 (45 haplotypes among 57 individuals) was from six KE populations (KE‐1, KE‐2, KE‐3, KE‐4, KE‐5, KE‐6) and was dominant in two subsurface KE populations (KE‐1, 17 haplotypes among 21 individuals; KE‐2, 17 haplotypes among 18 individuals). Lineage 2 (51 haplotypes among 61 individuals) was mainly from the surface populations in the KE region with three individuals from the subsurface KE‐2 population. Except for five individuals (five haplotypes) from the KE‐6 population, the other 67 individuals (56 haplotypes) of lineage 3 (61 haplotypes among 72 individuals) were from the SCS populations. The NJ tree combining COI data from global samples (Figure 3) indicated five clades, of which Clade 1–3 corresponded to lineage 1, lineage 3, and lineage 2 identified in the present study, respectively. Clade 4 was a subgroup composed of individuals from Spain and the Gulf of Mexico and clade 5 was the Chilean subgroup. Lineage 1 (i.e., clade 1) and lineage 2 (i.e., clade 3) could only be found in the KE.

The MSN constructed using COI haplotypes also revealed three distinct clades, and each clade was clustered mainly corresponding to the localities (Figure 2c). There were 59 bp of substitutions connecting clade 1 and clade 3, and 54 bp of substitutions connecting clade 2 and clade 3. Clade 1, clade 2, and clade 3 in the MSN correspond to lineage 1, lineage 2, and lineage 3 revealed by the NJ tree, respectively.

Pairwise *F*_ST_ values showed that there was large genetic differentiation between populations from SCS and populations from the KE region (0.522–0.917), and also between surface populations (KE‐3, KE‐4, KE‐5 and KE‐6) and subsurface populations (KE‐1 and KE‐2) from the KE region (0.380–0.709) (Figure 2d). AMOVA results revealed high and significant variations between groups (*Φ*_CT_ = 0.670, *p* < .001) and also within populations (*Φ*_ST_ = 0.673, *p* < .001) (Table 2). The results of IBD revealed no linear relationship between geographic distance and genetic distance for *L. anatifera* populations (COI: *R* ^2^ = 0.0697, *p* = .061) (Figure S1).

**TABLE 2:** Analysis of molecular variance statistics (AMOVA) for *Lepas anatifera* based on COI data.^a^ ^,^ ^b^

Source of variation	d.f.	Sum of squares	Variance components	Percentage of variation	Fixation indices
Among groups	2	2476.988	*V*_a_: 20.020	67.017	*Φ*_CT_: ***0*** *.**670***
Among populations within groups	6	69.933	*V*_b_: 0.090	0.302	*Φ*_SC_: ***0*** *.**009***
Within populations	181	1767.053	*V*_c_: 9.763	32.681	*Φ*_ST_: ***0*** *.**673***
Total	189	4313.974	29.873		

^a^ All individuals were divided into three groups according to the sampling latitude and depth: Group 1 (KE‐1, KE‐2), Group 2 (KE‐3, KE‐4, KE‐5, KE‐6) and Group 3 (SCS‐1, SCS‐2, SCS‐3).

^b^ d.f. is the degree of freedom; *Φ*_CT_, *Φ*_SC_ and *Φ*_ST_ is variation among groups, variation among populations within groups and variation within populations, respectively. Bold and italic values represent *p* < .05.

### 3.2 Historical demography

The mean net genetic distances (±SE) among the three lineages were shown as follows: 0.185 (0.045) between lineage 1 and lineage 3, 0.144 (0.035) between lineage 2 and lineage 3, and 0.167 (0.042) between lineage 1 and lineage 2. Using 1.4% to 2.33% Myr^−1^ (Knowlton & Weigt, 1998; Schubart et al., 1998) as mutation rates, the divergence of lineage 1 and lineage 2 was estimated to have occurred between 3.97 million years ago (Mya) and 6.61 Mya, and while lineage 2 and lineage 3 diverged about 3.09–5.14 Mya, lineage 1 and lineage 3 diverged about 3.58–5.96 Mya (Table S1).

Statistically significant negative Tajima's *D* and Fu's *F*_S_ of neutrality test as well as permutation tests with the SSD and HRI statistics (Table 3) consistently suggested that all lineages had experienced population expansions. At a mutation rate of 1.865% Myr^−1^, it was estimated that the expansion times of three lineages were 0.505, 0.251, and 0.266 Mya during the late Pleistocene interglacial cycles, respectively (Table 3). In the sequential mismatch distributions, all three lineages displayed a unimodal distribution. BSP analysis also indicated that three lineages had experienced demographic increases (Figure S2).

**TABLE 3:** Estimates of neural test and mismatch distribution for three lineages of *Lepas anatifera*.

Groups	Fu's *F*_*S*_	Tajima's *D*	Mismatch distribution					
			*θ*_0_	*θ*_1_	*p*(SSD)	*p*(HRI)	τ	Expansion time (Mya)
Lineage 1	−24.951**	−1.437*	0.004	46.094	0.270	0.550	8.783	0.505
Lineage 2	−25.320**	−2.077**	1.789	41.4062	0.450	0.840	4.363	0.251
Lineage 3	−25.722**	−1.922**	0.000	99999.000	0.210	0.250	4.623	0.266

*Note*: Tajima's *D* and Fu's *F*_*S*_ are shown, the value with one * represents *p* < .05 while two * represents *p* < .01. *θ*_0_ and *θ*_1_ are *θ* parameter before and after expansion; *τ* is mismatch distribution estimate; *p* values for the sum of squared deviations (SSD) and raggedness index (HRI) under the hypothesis of sudden expansion of each lineage are also shown.

### 3.3 Selection for temperature‐associated outlier SNPs

Raw GBS‐seq data of 116 samples have been submitted to GenBank (BioProject ID: PRJNA875021; BioSample accessions: SAMN30608385‐SAMN30608500). The BayeScan approach was applied to 15,238 SNPs (defined as “all SNPs”) retained after quality control and 425 SNPs were identified as outliers (all were under divergent selection for alpha >0) at the false discovery rate (FDR) threshold of 0.05. The left loci after removing the 425 loci under selection were further filtered by HWE and LD. After filtering HWE at *p* < .05 and LD at *r* ^2^ = .2, a total of 5180 SNPs were determined as “neutral SNPs.”

**FIGURE 4:**
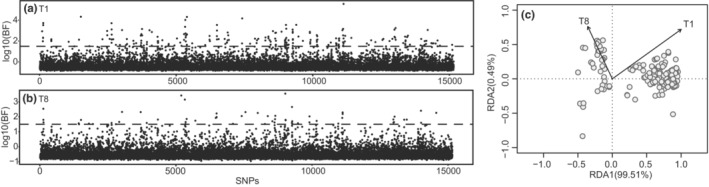
Manhattan plot and of genetic differentiation associated with temperature and redundancy analysis (RDA) based on temperature‐related SNPs. (a) The SNP allele frequency variation associated with T1; (b) The SNP allele frequency variation associated with T8. Black dashed lines mark lower thresholds of log10 (BF) = 1.5; (c) Redundancy analysis (RDA) based on temperature‐related SNPs. RDA axis 1 and axis 2 represent the proportion of the variance explained by T1 and T8, respectively. T1, Average water temperature in January from 2005 to 2017; T8, Average water temperature in August from 2005 to 2017.

The BAYENV analysis based on all 15,238 SNPs revealed that outlier loci could be screened based on both temperature variables (T1 and T8) (Figure 4a,b). With the criterion of log_10_ Bayes factor (BF) greater than 1.5, 226 candidate SNPs were identified as “outlier SNPs” associated with local temperature variables. Among these candidate SNPs, 213 and 59 SNPs were strongly correlated with the average temperature in January (T1) and August (T8), respectively, and 46 SNPs were correlated to both temperature variables. The 226 SNPs significantly associated with temperature variables were also identified as outliers by BayeScan.

Correlation coefficients between T1 and T8 was 0.89, and the variance inflation factor (VIF) values for the two predictors were below 5, indicating that multicollinearity between the two predictors should not be a problem for the model. For the RDA analysis based on the 226 SNPs associated with local temperature, the RDA model was globally significant (*p* = .001) with an adjusted coefficient of determination (*R*_adj_^2^) of 0.487, with T1 and T8 accounting for 55.36% and 44.72% of the total explained variance, respectively. The first two axes of the RDA accounted for 99.51% and 0.49% of the genetic variations, respectively (Figure 4c). The results of marginal ANOVAs for the RDA indicated that T1 and T8 were significant predictors of the putatively adaptive genetic variation (*p* = .001).

Among the 226 temperature‐related “outlier SNPs,” a total of 19 SNPs were annotated to five genes (Table 4). Among these annotated SNPs, 18 SNPs (SNP_38,653:16, SNP_38,653:142, SNP_38,655:94, SNP_83,533:238, SNP_83,532:19, SNP_83,532:66, SNP_83,533:182, SNP_83,532:106, SNP_ 83,532:166, SNP_83,533:82, SNP_83,630:100, SNP_83,631:144, SNP_101,157:48, SNP_101,160:157, SNP_101,157:51, SNP_101,157:133, SNP_101,160:72 and SNP_127,165:96) were identified by the temperature variable T1, and SNP_ 38,655:205 was identified by the temperature variable T8.

**TABLE 4:** Gene–environment associated SNPs and the putative genes.

SNPs ID	Gene name	Protein name	Putative function
101,157:48 101,160:157 101,157:51 101,157:133 101,160:72	CUO6_0	Cuticle protein 6	Enhancing stress resistance in the cuticle (Li & Denlinger, 2009).
83,630:100 83,631:144	Vg	Vitellogenin	Precursor of the egg‐yolk proteins providing nutrients during embryonic development (UniProtKB ID: A0A6A4VVG8).
38,653:16 38,655:205 38,653:142 38,655:94	AT1B	Sodium/potassium‐transporting ATPase α‐subunit	Catalyze the hydrolysis of ATP coupled with the exchange of sodium and potassium ions across the plasma membrane. (UniProtKB ID: A0A6A4WDD9)
83,533:238 83,532:19 83,532:66 83,533:182 83,532:106 83,532:166 83,533:82	(ORF names): FJT64_018671	RNA‐directed DNA polymerase	Involved in catalyzing DNA synthesis (Baltimore, 1970).
127,165:96	rpl31	60 S ribosomal protein L31	A structural constituent of ribosome; Involved in cytoplasmic translation (UniProtKB ID: A0A6A4WLN2).

Cuticle protein 6 coding by genes with SNP_101,157:48, SNP_101,160:157, SNP_101,157:51, SNP_101,157:133 and SNP_101,160:72 was potentially involved in enhancing stress resistance in the cuticle (Li & Denlinger, 2009). Vitellogenin that contains SNP_83,630:100 and SNP_83,631:144 provides nutrients during embryonic development (UniProtKB ID: A0A6A4VVG8). Sodium/potassium‐transporting ATPase α‐subunit which contains SNP_38,653:16, SNP_38,655:205, SNP_38,653:142, and SNP_38,655:94 can provide energy under stress (UniProtKB ID: A0A6A4WDD9). The proteins coding by genes containing SNP_83,533:238, SNP_83,532:19, SNP_83,532:66, SNP_83,533:182, SNP_83,532:106, SNP_83,532:166, and SNP_83,533:82 plays an important role in replication through catalyzing DNA synthetic and maintaining DNA stability (Baltimore, 1970). The 60 S ribosomal protein L31 coding by the gene containing SNP_127,165:96 was predicted to be a structural constituent of the ribosome and involved in cytoplasmic translation (UniProtKB ID: A0A6A4WLN2). More details of the putative functions of these proteins can be found in Table 4.

### 3.4 Population structure using GBS‐seq

Based on all 15,238 filtered SNPs, expected heterozygosity (*H*_e_) of populations, observed heterozygosity (*H*_o_), and nucleotide diversity (*π*_G_) ranged from 0.05676 to 0.06745, 0.04630 to 0.07909, and 0.05962 to 0.07064, respectively (Table 1). IBD based on neutral SNPs also revealed no linear relationship between geographic distance and genetic distance for *L. anatifera* populations (*R* ^2^ = .041, *p* = .620) (Figure S1).

**FIGURE 5:**
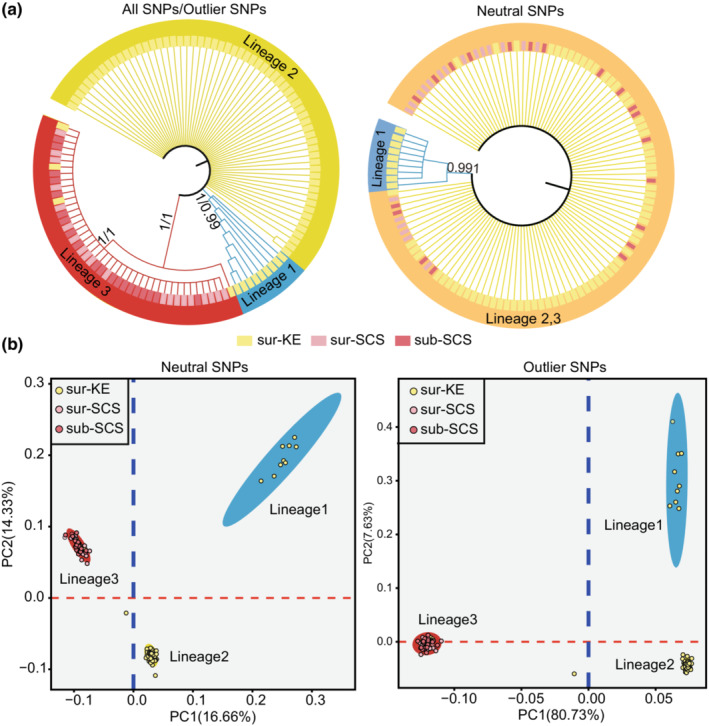
Phylogeographic structure of *Lepas anatifera* populations in Northwest Pacific based on GBS SNPs. (a) NJ tree of *L. anatifera* genetic lineages based on all SNPs, outlier SNPs and neutral SNPs of 116 samples collected from the Northwest Pacific; (b) Scatter diagram of the first two principal components (PCs) from analysis of neutral SNPs and outlier SNPs. sur‐KE, surface sites in the Kuroshio Extension (KE) region; sub‐KE, subsurface sites in the Kuroshio Extension (KE) region; sur‐SCS, surface sites in the South China Sea (SCS); sub‐SCS, subsurface sites in the South China Sea (SCS).

To test the possible effect of temperature‐based selection pressure on inferred genetic structure, population structure analysis was performed based on all SNP (15,238 SNPs), neutral SNP (5180 SNPs), and outlier SNP (226 SNPs) datasets, respectively. NJ tree constructed based on three SNP datasets also structured three lineages in *L. anatifera* populations except for the results of neutral SNPs with only two clusters (Figure 5a), which are consistent with the results of COI. Individuals in lineage 1 and lineage 2 were from the KE populations, and individuals in lineage 3 were mainly from the SCS populations with five individuals from the KE‐6 population. Three individuals in the KE‐6 population showed an exceptional trend: their COI sequences belonged to lineage 1 or lineage 3, but their GBS‐seq sequences were all grouped into lineage 2.

PCA analysis revealed that all populations were mainly divided into three groups, consistent with the three lineages identified by the NJ phylogenetic tree (Figure 4b). In the results of all SNPs (Figure S3) and neutral SNPs, the first principal component revealed genetic differentiation between lineage 1 and the other two lineages (lineage 2 and lineage 3), while PC2 largely separated lineage 2 and lineage 3. However, in the result of outlier SNPs, lineages in the KE region (i.e., lineage 1 and lineage 2) and lineage in the SCS (i.e., lineage 3) were separated by PC1 while lineage 1 and lineage 2 were separated by PC2. Besides, the first two principal components of the outlier dataset explained 88.63% of the total variation, much more than the variances explained by the first two principal components in all SNP datasets (55.77%) and the neutral SNP datasets (30.99%).

**FIGURE 6:**
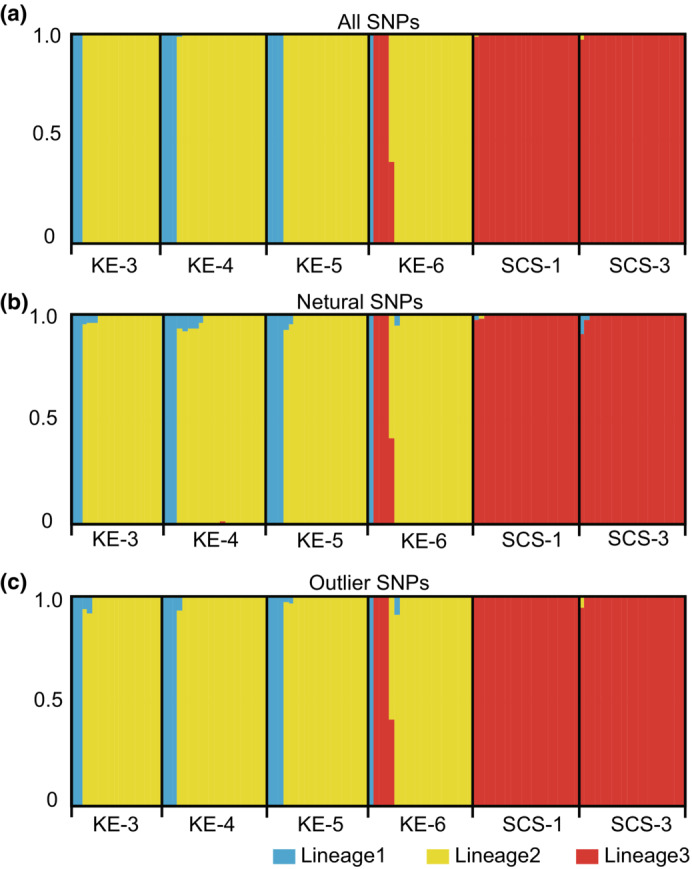
Admixture structure of SNP‐based co‐ancestries for *Lepas anatifera* samples (*K* = 3) using (a) all SNPs, (b) neutral SNPs, and (c) outlier SNPs.

The most suitable numbers of ancestral populations for the three datasets were three according to the K values with the lowest cross‐validation error (CV). The results of ADMIXTURE analysis based on three SNP datasets all revealed a genetic structure consistent with the results of PCA (Figure 6): first, all individuals from the SCS and three individuals from the KE region (i.e., KE‐6) formed one genetic group (i.e., lineage 3); second, while most of the other individuals from the KE region formed the other genetic group (i.e., lineage 2); finally, nine individuals formed the third genetic group (i.e., lineage 1).

## 4 DISCUSSION

Speciation or genetic divergence in the open ocean where presumably the absence of geographic barriers to gene flow has puzzled ecologists and evolutionary biologists. In the present study, we have found genetic differentiation between the SCS and the KE populations, and vertical genetic divergence in the KE populations of the pelagic barnacle *L. anatifera*. Our results also reveal that temperature, especially small‐scale thermal heterogeneity, is an important environmental factor in maintaining the high genetic diversity of pelagic species in the open oceans.

### 4.1 Genetic differentiation pattern of the pelagic barnacle *Lepas anatifera* in the Northwest Pacific

The goose barnacle *Lepas anatifera* exhibits high abundance worldwide (Young, 1990). In the present study, phylogenetic trees, pairwise *F*_ST_ values, genetic distances, ADMIXTURE results, and PCA plots all revealed that there were three divergent lineages of the pelagic barnacle *L. anatifera* with different geographical patterns in the northwest Pacific. Clear population structures revealed by both COI and neutral SNPs indicated the existence of historical genetic differentiation among the three lineages. The diverged time among the three lineages (at about 3–6 Mya) can date back to the Pliocene epoch, and all three lineages have experienced historical expansion during the late Pleistocene interglacial cycles. The temperature‐related outlier SNPs also distinguish the three lineages, especially in the PCA plot based on the outlier SNPs, and lineages from the KE region and SCS were largely separated.

The KE‐6 population in the Kuroshio Extension (KE) region contained haplotypes from all three lineages and potentially act as a transitional population between the Kuroshio Extension Region and the South China Sea. The existence of the lineage of SCS in KE‐6 could be potentially driven by the northward Kuroshio Current as what has been seen in other barnacles (Chan et al., 2007a, 2007b; Chang et al., 2017; Tsang et al., 2012). For example, the current‐driven northward range extension of the intertidal acorn barnacle *Hexechamaesipho pilsbryi* (Tsang et al., 2013).

The horizontal population differentiation or vertical differentiation of macrobenthos has been reported in previous studies (Bik et al., 2010; Ni et al., 2017; Pardo‐Gandarillas et al., 2018; Sun et al., 2017, 2022; Wang et al., 2022). However, studies of intraspecific population differentiation at both horizontal and vertical scales for epipelagic macrobenthos were still limited. In a previous study by Schiffer and Herbig (2016), the phylogeny of *L. anatifera* based on samples collected globally was investigated and three major groups of *L. anatifera* were revealed, inferring the allopatric differentiation of *L. anatifera* (Schiffer & Herbig, 2016). The NJ tree based on COI data showed that lineage 3 of the South China Sea identified in the present study corresponded to the global group from the study of Schiffer and Herbig (2016), suggesting the global distribution of lineage 3. There were no sequences downloaded from NCBI clustered with the lineage 1 and lineage 2 of the KE region, probably because the downloaded sequences were not comprehensive enough, and for the subsurface lineage 1, it was also likely due to the limitation of no subsurface sampling of the previous study. In the present study, the genetic distance between the surface lineage 2 of the KE region and lineage 3 of the South China Sea was smaller than that between lineage 1 and lineage 2, indicating higher genetic similarity despite geographical distance. The larger genetic differentiation between different vertical sites possibly was related to the different adaptability to the specific microhabitats (Han & Dong, 2020; Li, Tan, et al., 2021) and also implied the importance of small‐scale environmental heterogeneity in maintaining the genetic differentiation pattern. Due to the limitations of the sampling method (i.e., no fixed buoys or mooring system in the same station), in the present study, we cannot get samples from different water depths in the same station, which limits us from analyzing vertical differentiation in the same region. Hence, more samples from different vertical microhabitats should be collected for analysis in the future.

The barnacles *L. anatifera* in different populations face divergent thermal environments. This species usually thrives in tropical and subtropical waters where sea temperatures exceed 18–20°C (Hinojosa et al., 2006; Patel, 1959), and has been thought to survive in waters warmer than 15°C (Patel, 1959). The water temperatures in the SCS (0 m to 100 m in depth) are higher than 20°C in all seasons (Liu & Gan, 2017). In the surface sites in the KE region, temperatures are above 15°C all year round except for the KE‐3 where temperatures can be less than 15°C in the colder months. However, as for the subsurface sites in the KE region (i.e., KE‐1 and KE‐2), the water temperatures are below 15°C throughout the year (Wang et al., 2020). The low water temperature for the subsurface KE population (below 15°C) might act as an environmental selection factor filtering out other lineages and allowing lineage 1 to survive in the subsurface sites, leading to the different frequencies of lineage 1 and lineage 2 present in different depths. These results implied that temperature heterogeneity might be a physical barrier that prevents the gene flow between lineages with different thermal tolerances and plays a vital role in maintaining the genetic differentiation patterns of *L*. *anatifera* from the fixed buoys in the NWP.

The redundancy analysis based on the genotype–environment association method further confirmed the roles of temperature heterogeneity in maintaining the genetic differentiation of *L*. *anatifera* in the northwest Pacific. As previous studies reported (Han & Dong, 2020; Hu & Dong, 2022b), the genetic variations among populations of the mussel *Mytilus galloprovincialis* and the oyster *Crassostrea sikamea* were significantly correlated with the environmental temperatures. In the present study, the results of RDA analysis revealed that local temperature, especially the low temperature in January (e.g., T1) was a significant factor influencing the distribution patterns of putatively adaptive genetic variation among populations. Among the 19 candidate adaptive loci, cuticle protein 6 possibly plays an important role in enhancing stress resistance in the cuticle of *L. anatifera* (Li & Denlinger, 2009), and in the adaptation to local thermal environments.

Our results also indicated the potential possibility of hybridization and genetic introgression between different lineages. Both the SNPs‐based NJ tree and PCA plot indicated that the populations in the SCS were genealogically homogeneous, but there were mixed lineages present in the surface populations of the KE region. Particularly in the KE‐6 population, there was a sole population with a mixed distribution of three lineages. Furthermore, three individuals in the KE‐6 population showed an exceptional trend: their GBS‐seq belonged to lineage 1 or lineage 3, but their COI sequences were all grouped into lineage 2. Such topological inconsistency had been found in other species, such as the Japanese mantis shrimp *Oratosquilla oratoria* (Cheng & Sha, 2017) and the pantropical green seaweed genus *Halimeda* (Verbruggen et al., 2005). All these results have shown that secondary contact between high‐diverged lineages can cause hybridization and genetic introgression. Under the context of continuous global warming, the introgression from high‐temperature‐adapted lineage may bring genotypes that allow individuals adapted to lower temperatures to better adapt to the warming ocean (Hu & Dong, 2022a; Prada & Hellberg, 2021). Furthermore, incomplete lineage sorting could also lead to polytomies in the phylograms and the inconsistent lineage between mtCOI and nuclear genes (Cheang et al., 2010). Whether such a situation occurred between lineages of *L. anatifera* remains to be further tested.

### 4.2 The critical role of fixed substrate in investigating population connectivity in the open ocean

The placement of moorings and buoys in the KE region provides an ideal system for our studies of *L. anatifera* genetic differentiation patterns. The necessity of conducting the study either of free‐drifting debris in situ or the use of moorings as proxies had been mentioned in a recent study (Mesaglio et al., 2021). In the present study, *L. anatifera* specimens were sampled from fixed surface buoys and subsurface moorings placed in the northwest Pacific, allowing us to observe the divergence in the vertical direction. These artificial structures are helpful for our investigation of the distribution pattern and the corresponding formation and maintenance mechanisms of sessile macrobenthos in the open oceans.

Goose barnacles in the genus *Lepas* (family Lepadidae) are the most abundant and widespread biofouling taxa globally (Thiel & Gutow, 2005), with a planktonic larval stage of up to 2 months (Anderson, 1994; Darwin, 1851; Hinojosa et al., 2006). However, there were limited reports of goose barnacles distributed in deeper water layers. Due to the limitations of sample collection, most previous studies have defined the goose barnacles of *Lepas* as epipelagic rafters (Schiffer & Herbig, 2016). So far, most studies focused on the effects of floating objects on the long‐distance dispersal of the species and applied SST as an indicator for genetic differentiation. The present study emphasized the existence of genetic differentiation in the vertical direction and raised the importance of the vertical temperature profile as the maintaining force of genetic differentiation in the ocean. Therefore, micro‐geographic adaptation in different spatial scales should be valued to understand how species respond to their microclimates (Bates et al., 2018; Richardson et al., 2014).

## 5 CONCLUSION

The barnacle *L. anatifera* living in the Kuroshio Extension Region mainly diverged into surface and subsurface lineages, while another lineage of *L. anatifera* was in the SCS. The genetic differentiation was related to the difference in horizontal and vertical water temperatures, implying that both horizontal and vertical temperature heterogeneities play an important role in maintaining the genetic differentiation pattern in the open oceans.

## AUTHOR CONTRIBUTIONS

**Xiaonie lin:** Data curation (equal); methodology (equal); validation (equal); visualization (equal); writing – original draft (equal); writing – review and editing (equal). **Lisha Hu:** Conceptualization (equal); funding acquisition (equal); methodology (equal); visualization (equal); writing – original draft (equal); writing – review and editing (equal). **Zhaohui Chen:** Data curation (equal); methodology (equal); resources (equal); writing – review and editing (equal). **Yunwei Dong:** Conceptualization (equal); funding acquisition (lead); project administration (equal); writing – original draft (equal); writing – review and editing (equal).

## CONFLICT OF INTEREST STATEMENT

The authors have no conflict of interest to declare.

## Supporting information


Appendix S1

## Data Availability

The genetic data generated during the present study are available from the NCBI Sequence Read Archive (www.ncbi.nlm.nih.gov/genbank, BioProject ID: PRJNA875021; BioSample accessions: SAMN30608385‐SAMN30608500. GenBank accessions of mtDNA COI: OP215356‐OP215545).
