# Supplementary material for: Thermal heterogeneity is an important factor for maintaining the genetic differentiation pattern of the pelagic barnacle Lepas anatifera in the northwest Pacific
Source: Ecol Evol. 2023 Feb 22;13(2):e9843. doi: 10.1002/ece3.9843 (PMC9944158; doi:10.1002/ece3.9843)
Supplement: Supplementary file 1 — Appendix S1 [file ECE3-13-e9843-s001.docx]

**Appendix**

**Thermal heterogeneity is an important factor maintaining the genetic pattern of the pelagic barnacle *Lepas anatifera* in the northwest Pacific**

Xiao-Nie Lin^1#^, Li-Sha Hu^1,2#*^, Chao-Hui Chen^3,4^, Yun-Wei Dong^1,2*^

1 The Key Laboratory of Mariculture, Ministry of Education, Fisheries College, Ocean University of China, Qingdao, China

2 Function Laboratory for Marine Fisheries Science and Food Production Processes, Pilot National Laboratory for Marine Science and Technology, Qingdao, China

3 Frontier Science Center for Deep Ocean Multispheres and Earth System (FDOMES) and Physical Oceanography Laboratory, Ocean University of China, Qingdao, China

4 Qingdao National Laboratory for Marine Science and Technology, Qingdao, China

Table S1 The net average genetic distances between COI lineages and associated divergence time between three lineages of *Lepas anatifera* populations.

| Lineages | K2P genetic distance | Divergence time (Mya) |
| --- | --- | --- |
| Lineage 1 vs lineage 2 | 0.185±0.045 | 3.970~6.607 |
| Lineage 2 vs lineage 3 | 0.144±0.035 | 3.090~5.143 |
| Lineage 1 vs lineage 3 | 0.167±0.042 | 3.584~5.964 |


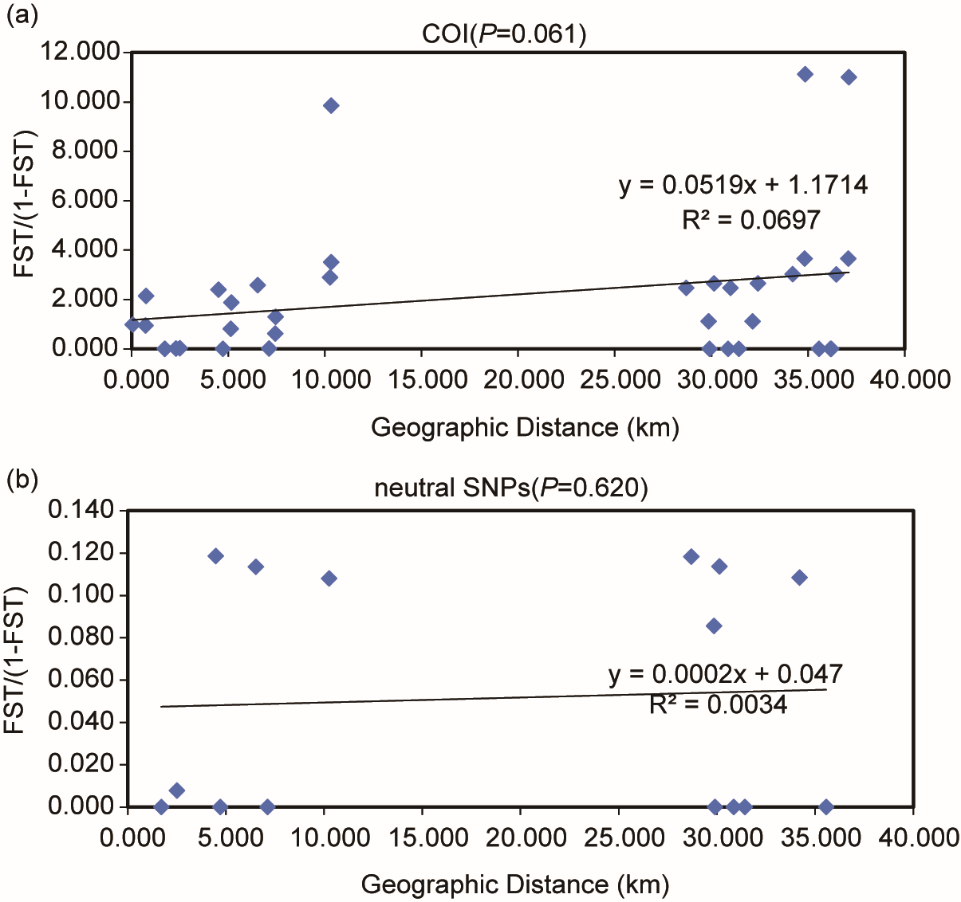


Figure S1 Isolation by distance patterns in *Lepas anatifera* in the Northwest Pacific, revealed by COI(a) and neutral SNPs(b).


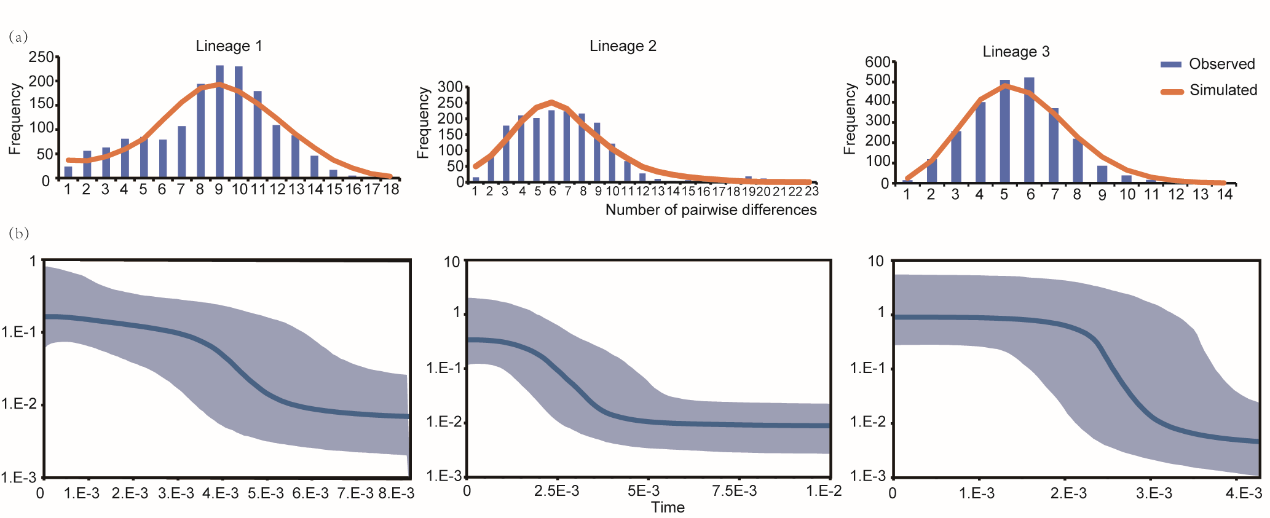


Figure S2 Mismatch distributions (a) and Bayesian skyline plots (b) for three genetic lineages of *Lepas anatifera* based on COI gene.


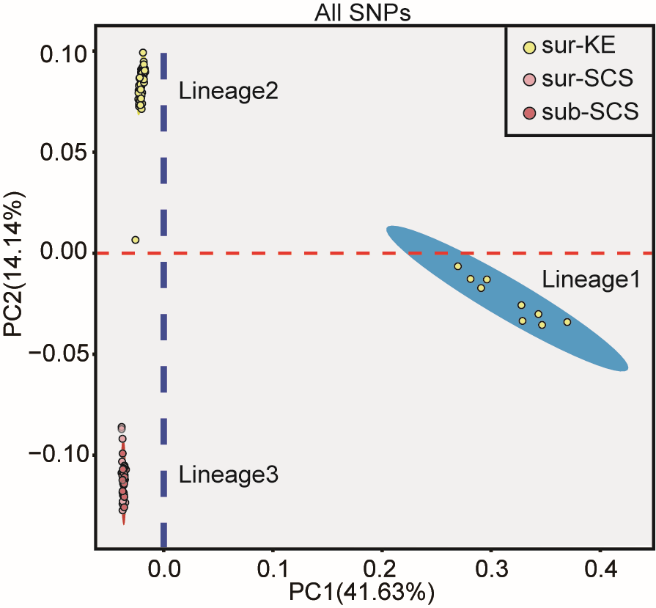


Figure S3 Scatter diagram of the first two principal components (PCs) from analysis of all SNPs.
